# Factors associated with changes in sexual behavior during Ramadan

**DOI:** 10.1192/j.eurpsy.2022.2073

**Published:** 2022-09-01

**Authors:** N. Sayari, A. Maamri, A. Hajri, R. Haoua, H. Zalila

**Affiliations:** Razi Hospital, Emergency And Outpatient Departement, manouba, Tunisia

**Keywords:** sexual behaviour, fasting, sexuality, Ramadan

## Abstract

**Introduction:**

Previous studies have shown that the month of Ramadan has a negative impact on the sexual life of fasters. Sexuality during Ramadan seems monotonous, rather poor, leaving little room for foreplay, sensuality and diversification of the sexual repertoire.

**Objectives:**

To examine the socio-demographic and religious factors associated with the change in the sexual lives of fasters

**Methods:**

A cross-sectional study was conducted among married Muslim volunteers in Tunisia. The data was collected with an anonymous self-completed questionnaire, one week before Ramadan (W-1) and the fourth week of Ramadan (W4).

**Results:**

We included 100 participants in this survey.The analytical study found a negative correlation between age and the frequency of coitus during Ramadan (r= -0.2, p= 0.04). The lenth of the marriage was associated with less communication about sexual satisfaction during Ramadan (P=0.01). Rural origin was associated with less tenderness (p=0.03) and shorter foreplay (p=0.03). Wearing the veil was associated with sexual abstinence in women during Ramadan (p=0.038) and not wearing it was associated with the cessation of oral sex (p=0.04). The practice of prayer was correlated to a lesser diversification of sexual positions (p=0.01) and to the withdrawal of certain sexual positions: posterior vaginal (p=0.01), lateral (p=0.02), Andromache (p=0.004).

**Conclusions:**

Changes in the expression of sexuality during Ramadan are not consistent with religious dictates. These findings suggest that the perception of sexuality and its practices are motivated by tradition and culture much more than religion.

**Disclosure:**

No significant relationships.

